# Spiritual Care Needs and Challenges Among Caregivers and Families of People with Neurodegenerative Diseases in Palliative and End-of-Life Care: A Scoping Review

**DOI:** 10.3390/brainsci16060611

**Published:** 2026-06-04

**Authors:** Enrico De Luca, Andreina Saba, Laura Bertarini, Antonio Brusini, Giovanna Artioli, Federica Dellafiore

**Affiliations:** 1 Department of Nursing and Midwifery, University of Birmingham, Birmingham B15 2TT, UK; 2 Department of Primary Care, Azienda USL—IRCCS di Reggio Emilia, 42122 Reggio Emilia, Italy; andreina.saba@studenti.unipr.it; 3 Department of General and Post-Acute Internal Medicine, University Hospital Modena, 41125 Modena, Italy; laura.bertarini1@studenti.unipr.it; 4 Modena Local Health Authority, AUSL Modena, 41125 Modena, Italy; antoniobrusini87@outlook.it; 5 Department of Life Health Sciences and Health Professions, Link Campus University, 00165 Rome, Italy; g.artioli@unilink.it (G.A.); f.dellafiore@unilink.it (F.D.)

**Keywords:** spiritual care, palliative care, neurodegenerative disease, caregivers, scoping review

## Abstract

**Highlights:**

**What are the main findings?**

- Spiritual needs among caregivers and health professionals in neurodegenerative palliative care are relational and meaning-focused, extending beyond religion.
- A persistent misalignment between caregivers’ spiritual experiences and healthcare system responses, with spiritual care remaining fragmented and inconsistently integrated into practice.

**What are the implications of the main findings?**

- There is a clear need for integrated, caregiver-inclusive and culturally responsive models of spiritual care across end-of-life neurodegenerative disease settings.
- Greater conceptual clarity and system-level approaches to spiritual care are required to inform healthcare professions’ education, practice development and future research.

**Abstract:**

**Background/Objectives:** Spirituality is increasingly recognised as a core dimension of holistic and palliative care. Neurodegenerative diseases such as dementia, amyotrophic lateral sclerosis and Parkinson’s disease involve prolonged trajectories of loss, uncertainty and relational change, which may heighten spiritual and existential needs for patients, particularly among those involved in caregiving, such as family caregivers and, to a lesser extent, healthcare professionals. However, evidence on how spirituality is understood, experienced and addressed within neurodegenerative palliative care remains fragmented and conceptually heterogeneous. This scoping review aimed to map the literature on caregivers’ spiritual needs and challenges. **Methods:** A scoping review was conducted in accordance with the Joanna Briggs Institute (JBI) methodology for scoping reviews and the Preferred Reporting Items for Systematic Reviews and Meta Analyses extension for Scoping Reviews (PRISMA ScR). Searches were conducted across PubMed, Cumulative Index to Nursing and Allied Health Literature (CINAHL), APA PsycINFO, and Scopus, with no date or geographical restrictions. Grey literature was searched through Google Scholar and relevant organisational and policy sources in the field of palliative care and spirituality. Reference list screening of included studies and relevant reviews was also conducted. Quantitative, qualitative, and mixed methods studies published in English or Italian were included. **Results:** Twenty-four studies published between 2007 and 2025 were included. Findings were organised into three interconnected domains: spiritual needs, spiritual processes and spiritual care. Spirituality emerged as a dynamic, relational and context-dependent dimension of caregiving, encompassing meaning, identity, connection and coping with vulnerability and loss. Spiritual needs and processes were widely described, while spiritual care was inconsistently recognised within healthcare systems. Conceptual ambiguity, under-representation of end-of-life dementia and cultural imbalances were evident. The evidence predominantly focused on family caregivers, with limited representation of healthcare professionals. **Conclusions:** This scoping review highlights a persistent gap between caregivers’ lived spiritual experiences and system-level responses in neurodegenerative palliative care in caregiving contexts globally. The findings support integrated, caregiver-inclusive and culturally responsive approaches to spiritual care.

## 1. Introduction

Spirituality has long been recognised as an integral dimension of human life, yet its meaning and expression vary widely across cultures, traditions and personal worldviews [1]. In this review, spirituality is understood as a broad and multidimensional construct encompassing meaning, purpose, identity, connection, and, where relevant, transcendence. Contemporary health organisations, including the World Health Organization, identify spirituality as a fundamental component of well-being and holistic care [2]. Rather than being limited to religious belief, spirituality encompasses broader experiences of meaning, purpose, identity, connection and transcendence [3,4,5]. For many people, especially those facing serious illness, spirituality plays a central role in coping with uncertainty, maintaining hope and making sense of profound life transitions [6,7]. In palliative care, spirituality has gained increasing prominence as an essential aspect of compassionate and person-centred care [8]. Frameworks such as the European Association for Palliative Care (EAPC) definition emphasise spirituality as a dynamic dimension of human experience expressed through relationships with self, others, nature or the sacred [9].

This perspective reflects the growing recognition that spirituality may influence how patients understand and live with life-limiting illness, and how families navigate the emotional and existential challenges of caregiving and bereavement. Neurodegenerative conditions such as dementia, amyotrophic lateral sclerosis (ALS) and Parkinson’s disease involve progressive loss, shifting identities, altered relationships and prolonged periods of uncertainty [10]. These trajectories often heighten questions about meaning, suffering, autonomy and connection. While the literature highlights that people with neurodegenerative conditions frequently express spiritual needs related to hope, purpose, reconciliation and inner peace [11,12,13] emerging research has increasingly drawn attention to the needs of family caregivers and healthcare professionals as well. Caregivers often shoulder not only physical and emotional responsibilities but also the spiritual burden of witnessing decline, anticipating loss and navigating changing family roles [14,15]. Healthcare professionals, who routinely encounter suffering, ethical dilemmas and end-of-life transitions, likewise describe a need for space, reflection and support in addressing existential aspects of care [16,17]. Importantly, the spiritual dimension of caregiving is often hidden. Family members may mask emotional or existential distress to “stay strong,” manage complex care responsibilities or protect the patient from additional worry [18].

This internalised burden can obscure unmet needs that go unrecognised by health services [19]. Evidence increasingly shows that such unaddressed distress can escalate into severe psychological consequences [20,21]. Notably, a recent analysis of cases in England and Wales identified caregiver-perpetrated homicides over a five-year period, often associated with perceived unbearable caregiving strain or attempts to “end suffering.” These cases were not formally classified as euthanasia, but rather as extreme outcomes associated with caregiver burden and distress. Beyond these extreme events, research shows that up to 26% of family caregivers of people with dementia report thoughts of suicide, highlighting the seriousness of caregiver distress and the urgent need for more holistic support, including attention to spiritual well-being [22,23]. These findings suggest that the spiritual and existential dimensions of caregiving remain significantly underestimated in both research and healthcare policy. At the same time, the concept of spirituality within neurodegenerative palliative care remains broad and variably defined across studies, making it challenging to understand how spiritual needs are conceptualised, assessed or supported in different contexts [9]. Much of the existing literature focuses on patients, with fewer studies exploring the interconnected needs of caregivers and professionals or examining how spirituality is embedded within broader care systems and organisational cultures [24]. While both family caregivers and healthcare professionals experience spiritual and existential challenges in this context, the caregiving experience is predominantly embodied by family members, who carry the long-term relational and emotional burden of care.

Previous evidence syntheses on spirituality in neurological and end-of-life care include both systematic and scoping reviews; however, they remain heterogeneous in scope and focus. Existing reviews have primarily examined spirituality in specific disease populations, such as stroke [25] or dementia [26], or within broader neuropalliative and professional care frameworks where caregivers were not the primary focus of analysis [27,28]. Across these reviews, spirituality in caregiving contexts is typically explored within single diagnostic groups, treated as a secondary dimension, or embedded within patient- or profession-focused perspectives. To date, no review has comprehensively mapped how spirituality is conceptualised, experienced, and addressed across caregiving roles within neurodegenerative palliative and end-of-life care contexts.

Given the conceptual breadth and heterogeneity of spirituality, alongside the diversity of populations, contexts, and study designs in neurodegenerative palliative care, a scoping review was considered the most appropriate methodology to map existing evidence, clarify key concepts, and identify gaps in the literature. The lack of conceptual clarity and systems-level perspective limits the ability to synthesise knowledge and to identify where meaningful support is most needed. Addressing these gaps requires an approach capable of mapping the scope, range and conceptualisation of the literature, rather than evaluating intervention effectiveness. A scoping review is therefore particularly appropriate to explore the heterogeneity of study designs, populations and conceptual definitions, and to identify gaps in knowledge and future research directions.

## 2. Materials and Methods

### 2.1. Study Design

A scoping review was conducted in accordance with the Joanna Briggs Institute (JBI) methodology for scoping reviews [29,30]. The conduct and reporting of the review followed the PRISMA extension for Scoping Reviews (PRISMA-ScR) guidelines [31]. A protocol for the review was developed a priori and published previously [32].

The protocol has been registered with the Open Science Framework https://doi.org/10.17605/OSF.IO/X9275, and the review was undertaken in winter 2025 and completed in April 2026.

### 2.2. Developing the Research Question

The review question was developed using the Population–Concept–Context (PCC) framework recommended by the Joanna Briggs Institute for scoping reviews [29]. The review question was: “What is known from the existing literature about the spiritual needs and challenges experienced by individuals involved in caregiving roles (primarily family caregivers and, where available, healthcare professionals) caring for adults with end-stage neurodegenerative diseases?”

### 2.3. Search Strategy

This scoping review examined a relatively new and evolving topic. To capture a broad range of evidence, no publication date restrictions were applied. Databases were selected for relevance and coverage across biomedical, nursing, psychological, and multidisciplinary literature.

Electronic searches were conducted in PubMed, Cumulative Index to Nursing and Allied Health Literature (CINAHL), APA PsycINFO, and Scopus. Grey literature was searched through Google Scholar and by screening relevant organisational and policy sources in the field of palliative care and spirituality, including the websites of the Italian Society of Palliative Care and the European Association for Palliative Care. Citation checking of included studies and relevant reviews was also undertaken to identify additional eligible records.

The search strategy was developed collaboratively by the research team in consultation with an experienced academic librarian from the University of Birmingham library service. The initial search strategy was developed for PubMed and subsequently adapted for the other databases. Full search strategies for all databases are provided in Supplementary Material S1.

The search was conducted for title and abstract fields, with no date restrictions and limited to English and Italian language. Search strategies for the other databases were adapted from the PubMed strategy using database-specific controlled vocabulary and syntax. All records were managed using Zotero (see https://www.zotero.org/) which supported reference organisation and full-text retrieval.

### 2.4. Inclusion and Exclusion Criteria

This scoping review applied the Joanna Briggs Institute Population–Concept–Context (PCC) framework [30]. The population of interest primarily consisted of family caregivers (relatives and informal carers providing unpaid care), with healthcare professionals included where relevant. The concept of interest included spirituality, spiritual needs, spiritual challenges, and spiritual care within neurodegenerative palliative and end-of-life care contexts. Primary studies employing qualitative, quantitative, and mixed-method designs were eligible for inclusion. No date or geographical restrictions were applied. Studies published in English or Italian and addressing major neurodegenerative diseases were included. Studies were excluded if they focused exclusively on patients without considering caregiving roles, did not address spirituality or spiritual care, or were unrelated to neurodegenerative palliative or end-of-life care contexts. Reviews, editorials, commentaries, conference abstracts, study protocols, and other non-empirical publications were also excluded.

Therefore, primary studies employing qualitative, quantitative, and mixed-method designs on spiritual care in neurodegenerative palliative care were included. No date or geographic limits were applied. English- and Italian-language studies covering major neurodegenerative diseases were included. Previously published reviews, including systematic and scoping reviews, were not eligible for inclusion in the final synthesis, as this review focused exclusively on primary studies. However, relevant reviews identified during the screening process were examined for contextual understanding and backward reference list screening (snowballing) to support the identification of additional eligible primary studies. In particular, two recent and accurate scoping reviews were used for citation and reference checking only and were not included as units of analysis in the final synthesis [33,34].

### 2.5. Study Selection and Data Extraction

All records identified through the search strategy were imported into Rayyan [35] and screened in two stages: title/abstract screening followed by full-text assessment. Both title/abstract screening and full-text eligibility assessment were conducted by four reviewers (AS, EDL, AB, and LB) working in pairs using Rayyan. Screening was conducted in pairs to ensure feasibility and consistency given the volume of records and varying reviewer experience. Blinding was enabled during title and abstract screening to minimise potential selection bias. Disagreements between reviewers were resolved through discussion during regular inter-screening meetings, and unresolved conflicts were adjudicated by a fifth reviewer (GA). The overall screening and selection process is reported in the PRISMA-ScR flow diagram.

Data extraction was conducted independently by four reviewers (AS, EDL, AB, and LB) using a data extraction table developed by the research team in line with the review objectives and eligibility criteria. The extraction tool was piloted on three studies and refined prior to full data extraction. Extracted data included study characteristics, populations, contexts, methodology, and key findings related to spirituality in caregiving contexts. Discrepancies in data extraction were resolved through discussion, with unresolved disagreements adjudicated by a third reviewer (GA).

### 2.6. Data Synthesis

Data synthesis aimed to provide a structured and interpretative mapping of the evidence, consistent with the objectives of a scoping review. Given the inclusion of heterogeneous study designs (quantitative, qualitative, and mixed-methods), an integrated narrative and thematic synthesis approach was adopted. Initially, extracted data were organised descriptively to summarise study characteristics, populations, and contexts. Quantitative findings were synthesised narratively, focusing on the direction and nature of reported associations without statistical pooling. Qualitative findings were analysed thematically using an inductive approach to identify recurrent patterns related to spiritual needs, challenges, and care practices.

Findings from different study designs were subsequently interpreted together at a conceptual level through iterative team discussions, allowing the identification of overarching analytical domains that conceptualised spirituality as a multidimensional and dynamic phenomenon across caregiving contexts. This approach is consistent with JBI guidance for scoping reviews, which emphasises mapping, categorisation, and conceptual clarification of heterogeneous evidence rather than quantitative aggregation [29,30].

## 3. Results

**Figure 1:**
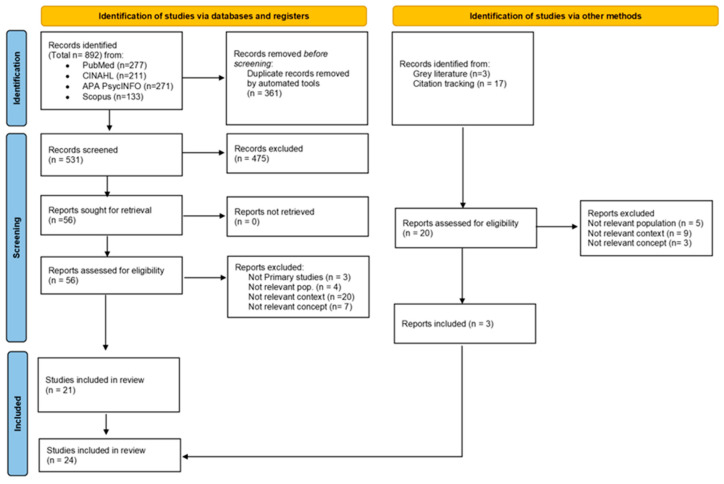
PRISMA 2020 flow diagram of the study selection process for this scoping review, adapted from Page et al. [36].

The database search yielded 892 records, of which 361 duplicates were removed, leaving 531 records for title and abstract screening. After this phase, 56 reports (full-text articles) were assessed for eligibility. Reports not meeting the predefined inclusion criteria were excluded, resulting in 21 eligible studies. An additional 20 records were identified through reference checking and grey literature searches, and 3 of these met the inclusion criteria following full-text assessment. In the present review, each included report corresponded to a distinct study; therefore, the term “study” is used throughout the manuscript for readability and consistency. In total, 24 studies were included in the final synthesis, as illustrated in the PRISMA flow diagram (Figure 1), encompassing a broad chronological span (2007–2025). Of these, 13 were published after 2020, indicating an increasing research focus on the spiritual dimensions of caregiving in advanced neurodegenerative conditions.

### 3.1. Characteristics of the Studies

#### 3.1.1. Countries of Publication

Most studies were conducted in the Global North. The United States contributes the highest number of studies [15,37,38,39,40,41,42,43,44,45,46], followed by Canada [47,48], Germany ([49], partial inclusion in [50]), the Netherlands [51,52,53], Australia [54,55], and Sweden [56]. Together, these countries constitute approximately two thirds of the included evidence, highlighting a predominantly Euro-American perspective. By contrast, only three studies derive from non-Western contexts: Brazil [57], Indonesia [58], and India [59]. These studies articulate conceptions of spirituality that are culturally and geographically grounded (Table 1).

#### 3.1.2. Research Methodologies

In terms of study design, the evidence base is heterogeneous and incorporates a substantial number of qualitative and mixed-methods studies. Ten studies employed purely qualitative methodologies, including phenomenology, thematic analysis, ethnography, and qualitative descriptive designs [15,38,40,41,53,55,56,57,58,59]. In addition to these, five studies adopted mixed-methods designs, combining qualitative interviews or open-ended components with quantitative assessment tools [42,44,45,48,54]. The remaining nine studies employed primarily quantitative approaches, such as cross-sectional surveys, longitudinal designs, or correlational analyses [37,39,43,47,49,50,51,52] (Table 1).

#### 3.1.3. Studies Participants and Types of Neurodegenerative Diseases

The population considered is predominantly composed of family caregivers, who represent the primary focus of the included studies (22 out of 24). In contrast, healthcare professionals are minimally represented, with only two studies specifically addressing their perspectives. Sample sizes varied substantially, with caregivers recruited from home-based care settings, long-term care facilities, and mixed home–hospital contexts. A smaller subset of studies (2) focused on healthcare professionals, mainly physicians and nurses working in long-term care services or neurology units. Overall, the studies addressed a wide range of neurodegenerative conditions, with dementia, amyotrophic lateral sclerosis (ALS), and Parkinson’s disease being the most represented (Table 1).

#### 3.1.4. Assessment Tools for Spirituality and Their Associations

The quantitative and mixed-methods studies adopted different tools to assess spirituality. The Functional Assessment of Chronic Illness Therapy–Spiritual Well-Being (FACIT-Sp) emerged as the most frequently used instrument [37,39,42,47]. The Spiritual Needs Questionnaire (SpNQ) provided a complementary perspective by identifying unmet spiritual needs and linking them with psychosocial factors such as loneliness among ALS patients and caregivers [49]. The Caring Ahead Questionnaire and the Carers’ Alert Thermometer integrated spirituality within broader evaluations of caregiving stress, preparedness, and emotional strain [48,54]. Across studies, spirituality was consistently examined in relation to emotional distress, caregiver burden, preparedness, decision-making, and quality of life, reflecting researchers’ efforts to situate spiritual well-being within broader psychosocial and clinical frameworks [38,39,40,43,45,49] (Table 1).

### 3.2. Thematic and Conceptual Organisation of Findings

The findings of this scoping review were synthesised using a convergent narrative and thematic approach, integrating evidence from quantitative, qualitative, and mixed-methods studies. In line with the analytical strategy described in the Methods, data were first examined descriptively and then interpreted at a conceptual level to identify patterns across studies and study designs. Through an iterative process of coding, comparison, and team-based discussion, findings were progressively organised into higher-order categories. This process enabled the integration of quantitative results—considered in terms of direction and nature of associations—with qualitative insights into lived experiences and meaning-making processes.

The synthesis resulted in the identification of three interconnected analytical domains: (1) spiritual needs, (2) spiritual processes, and (3) spiritual care. These domains were developed to move beyond a purely descriptive summary and to conceptualise spirituality as a multidimensional and dynamic phenomenon, encompassing what caregivers need, how spirituality is experienced and enacted, and how healthcare systems respond—or fail to respond—to these dimensions. Within this overarching framework, seven thematic areas were identified to provide a more granular, data-grounded representation of the evidence: (1) nature and dimensions of spiritual needs; (2) spiritual challenges and sources of distress; (3) spirituality as a coping and transformative resource; (4) factors associated with spiritual well-being; (5) spiritual care interventions and support strategies; (6) barriers and gaps in spiritual care provision; and (7) contextual influences on spiritual needs and care (Table 2).

#### 3.2.1. Spiritual Needs: What Caregivers Require

This domain captures the range of existential, relational, and, in some cases, religious needs expressed by family caregivers, corresponding primarily to the theme of nature and dimensions of spiritual needs. Across the included studies, spiritual needs extended well beyond organised religious practices (e.g., participation in religious services) and included the search for meaning and purpose, inner peace, hope, comfort, emotional closeness, connection with others, and, where relevant, a transcendent dimension. These needs also encompassed the desire to maintain rituals, preserve identity, and feel supported in facing illness, dying, and loss. Studies using structured measures such as the Spiritual Needs Questionnaire (SpNQ) and FACIT-Sp conceptualised spirituality across domains including meaning, peace, faith, existential needs, and generativity [37,39,47]. Qualitative evidence further deepened this perspective, highlighting caregivers’ need to sustain hope, preserve a sense of personal value, maintain relational connectedness, and feel spiritually accompanied throughout the illness trajectory [38,55,57]. In specific contexts such as dementia care, spiritual needs also included preserving rituals, traditions, prayer, and meaningful end-of-life practices [48,51]. Notably, several studies emphasised that spirituality could provide comfort and strength even in the absence of explicit religious identification [40,56]. Overall, this domain highlights that spiritual needs represent a core dimension of the caregiving experience, closely intertwined with meaning, identity, and relational continuity.

#### 3.2.2. Spiritual Processes: How Spirituality Operates Within Caregiving

This domain captures spirituality as a dynamic process shaped by tension, adaptation, and transformation and integrates three thematic areas.

#### 3.2.3. Spiritual Challenges and Sources of Distress

Spirituality emerged as a vulnerable dimension, particularly under conditions of degenerative illness and end-of-life care needs. Caregivers frequently experienced anticipatory grief, uncertainty, loneliness, emotional exhaustion, and difficulties in maintaining existential meaning, hope, or inner peace. In ALS and MND, spiritual distress was associated with loss of meaning, isolation, fear, guilt, and existential disorientation [44,56,57,59]. In dementia care, distress often emerged during the farewell phase, characterised by grief, uncertainty, and concerns about unmet relational or spiritual needs [38,41,45,51]. Quantitative evidence further indicated that depression, anxiety, symptom burden, and poor communication may undermine spiritual well-being [37,39,47]. Importantly, some studies suggested that spiritual distress often remains under-recognised and insufficiently assessed, despite its substantial impact [42,44].

#### 3.2.4. Spirituality as a Coping and Transformative Resource

Alongside vulnerability, spirituality functioned as an adaptive and transformative resource. Caregivers described spirituality as supporting resilience, emotional endurance, meaning-making, and existential reorientation. Qualitative studies showed that spirituality enabled caregivers to reinterpret suffering, sustain hope, remain connected to their relative, and continue engaging with life despite anticipated or actual loss [40,56,57]. This process could involve prayer, faith, reflection, inner strength, trust in a higher power, or a more personal search for meaning [39,40,54]. In chronic conditions such as dementia and Parkinson’s disease, spirituality supported coping with long-term burden, grief, and caregiving demands [44,45,59]. In some cases, caregivers described a transformative shift, moving from distress toward a deeper understanding of their role and of the illness experience itself [57].

#### 3.2.5. Factors Associated with Spiritual Well-Being

Spiritual processes were further shaped by a range of interacting psychological, relational, and clinical factors. Higher spiritual well-being was associated with greater preparedness, effective communication, lower levels of anxiety and depression, reduced burden, stronger resilience, social support, and hope [37,39,43,46,47]. Conversely, caregiver distress and patient-related factors—such as symptom burden and disease trajectory—could negatively influence spiritual well-being. In ALS caregiving, variables such as loneliness and spiritual faith influenced how caregivers approached complex decisions, including those related to end-of-life [49,50]. In dementia, preparedness for end-of-life, including its spiritual dimension, emerged as a key factor [41,48].

#### 3.2.6. Spiritual Care: How Systems Meet—Or Fail to—Caregivers’ Needs

This domain integrates the themes spiritual care interventions and support strategies, barriers and gaps in spiritual care provision, and contextual influences on spiritual needs and care, and focuses on how spirituality is addressed within healthcare systems.

#### 3.2.7. Interventions and Support Strategies

The literature indicates that spiritual care for caregivers is feasible but inconsistently implemented. Reported strategies included spiritual needs assessment, active listening, communication about meaning and values, support for difficult conversations, referrals to chaplains or spiritual counsellors, psychological support, and group-based interventions [44,49,51,54]. Structured tools such as the SpNQ, FACIT-Sp, CAT, and the Caring Ahead questionnaire highlighted the potential of more systematic approaches to identifying needs and supporting preparedness [48,49,54]. In some contexts, such as nursing homes, spiritual care included religious rites, visits from spiritual counsellors, and informal support from staff, although these were not consistently integrated into care pathways [52,53]. A chaplain-led intervention demonstrated acceptability and perceived usefulness, particularly in supporting reflection, communication, and emotional processing [42].

#### 3.2.8. Barriers and Gaps in Provision

Within this review, spiritual care was frequently described as underdeveloped, fragmented, and dependent on individual initiative rather than structured systems. Key barriers included lack of training, limited confidence among professionals, absence of systematic assessment, poor integration into care processes, and unclear professional roles, particularly regarding who is responsible for assessing and addressing spiritual needs within the care team [51,52,53]. Caregivers often reported unmet spiritual needs, particularly during critical phases such as end-of-life and bereavement [51,55]. Moreover, observational studies suggested that, although professionals may acknowledge the importance of spirituality, it is often not explicitly addressed or documented in practice [53]. As a result, caregivers frequently rely on personal coping strategies rather than formal support systems, which is generally described in the literature as a limitation or gap in care provision [38,41,49].

#### 3.2.9. Contextual Influences

The provision and experience of spiritual care were strongly shaped by cultural, organisational, geographical, and ethical contexts. The meaning of spirituality varied across settings, with some studies emphasising non-religious dimensions such as inner peace and generativity, and others highlighting faith, ritual, and religious traditions [44,49,57]. Factors such as rurality, access to healthcare, and local cultural norms influenced both needs and support [37]. Legal and ethical frameworks also played a role, particularly in relation to end-of-life decision-making, as shown in cross-country comparisons [50]. Organisational context—including differences between home care, nursing homes, and specialised services—further shaped the availability and nature of spiritual care [48,51,52,53].

## 4. Discussion

This scoping review mapped spiritual care needs among individuals involved in caregiving roles, with a predominance of evidence focusing on family caregivers and more limited representation of healthcare professionals. This imbalance in the evidence base should be explicitly acknowledged, as it shapes the interpretation of findings and highlights the centrality of family caregiving in this context. The findings were organised into three interconnected domains—spiritual needs, spiritual processes, and spiritual care—conceptualising spirituality as a dynamic, relational and context-dependent dimension of caregiving rather than a discrete or belief-based construct. Together, these domains highlight key points for discussion that reflect pressing and under-recognised caring needs within contemporary palliative and end-of-life care.

### 4.1. Caregiver-Centred Spirituality and Systemic Inequalities

Across the findings, spirituality emerged primarily through the experiences of family caregivers, reflecting the centrality of informal care in neurodegenerative disease trajectories [18,26]. Rather than being confined to religious belief, spirituality was consistently framed as a relational and existential resource tied to meaning, identity and continuity across the illness trajectory [15,51,52,60]. The predominance of caregiver-focused perspectives, alongside the relative absence of healthcare professional accounts, suggests that spirituality is often experienced as a personal and relational responsibility rather than a structured component of clinical care. This pattern aligns with broader observations that spiritual concerns in neurodegenerative palliative care are largely negotiated within family contexts, particularly in home-based and long-term caregiving arrangements rather than as a structured clinical competence embedded within healthcare services [26,49].

### 4.2. Misalignment Between Lived Spiritual Needs and System Responses

In addition, the review highlights important questions regarding equity and availability of support for caregivers. While the findings identified a range of spiritual care interventions and support strategies, their implementation appeared fragmented and inconsistent. A clear misalignment emerged between the depth and persistence of caregivers’ spiritual needs and the limited, often ad hoc, ways in which healthcare systems respond [46,47,48,61]. Spiritual care is frequently dependent on individual initiative, informal support, or specialist referral rather than being embedded within routine care pathways. As a result, caregivers often relied on personal coping strategies in the absence of healthcare system support [33,44]. This gap underscores the need to move beyond recognising spirituality as important toward integrating it as a core component of caregiver-inclusive palliative care [6,9].

### 4.3. Conceptual Ambiguity Between Spirituality, Religiosity and Psychological Well-Being

Persistent conceptual ambiguity across studies—particularly the conflation of spirituality with religiosity or psychological well-being—complicates assessment and intervention development. Similar concerns have been raised across palliative and nursing literature, where inconsistent definitions limit both clinical applicability and research comparability [9,11,24]. While the diversity of tools and definitions reflects the multidimensional nature of spirituality, it also risks reframing spiritual distress solely as emotional burden, thereby obscuring distinct existential needs [6]. Greater conceptual precision is therefore essential to support meaningful assessment, education and intervention design across care settings.

### 4.4. Cultural and Geographical Differences

The predominance of studies originating from the Global North, particularly the United States, raises important questions about how spirituality in neurodegenerative end-stage caregiving is conceptualised and whose understandings are privileged within the evidence base. This distribution likely reflects both the dominance of Western palliative care models and the prominence of individualised, often non-religious, conceptualisations of spirituality in Euro-American contexts [6,9,16]. As a result, spirituality is frequently framed as a personal, internal experience, potentially obscuring relational, communal and faith-embedded dimensions that may be more salient in other cultural settings. In contrast, evidence from Global South contexts—although limited—suggests that spirituality is often more explicitly integrated into religious practice, community life and collective meaning-making, and less frequently conceptualised as a discrete or individual domain [52,53,54]. However, even in these contexts, reflective engagement by healthcare professionals with their own spirituality was often limited, reinforcing the persistence of conceptual ambiguity across cultural settings. Taken together, these patterns raise critical questions for future research. To what extent do dominant Western frameworks shape how spiritual needs are identified, measured and prioritised in neurodegenerative care? How might alternative, culturally embedded understandings of spirituality challenge prevailing assumptions about individual coping, professional roles and care delivery? Addressing these questions requires greater inclusion of research from under-represented regions, alongside methodological approaches that attend to spirituality as a culturally situated, relational and system-embedded phenomenon.

## 5. Implications for Practice, Education and Future Research

### 5.1. Spiritual Care as a Shared, Embedded Component of Everyday Practice

Our scoping review reinforces the need to reconceptualise spiritual care as a shared responsibility across healthcare teams, rather than a delegated or specialist activity [55,59]. Healthcare professionals involved in neurodegenerative and palliative care should be supported to recognise and engage with spiritual needs, acknowledging that the relevance of spirituality may vary across individuals and contexts. Integrating spiritual care into routine clinical practice—rather than positioning it as an optional or referral-based intervention—may reduce the invisibility of spiritual distress and promote more holistic, caregiver-inclusive care [3,4,9,59].

### 5.2. Education and Training Beyond Religiosity, Grounded in Relational and Existential Care

The findings highlight the importance of education and training that should equip healthcare professionals with skills to identify existential, relational and meaning-based concerns, including attentive listening, presence, and communication about values and uncertainty [57,58,62]. Embedding spiritual care competencies within undergraduate curricula, continuing professional development and palliative care education may enhance professional confidence and reduce reliance on informal or ad hoc approaches [8,12,48]. Such training is particularly relevant in settings where caregivers carry prolonged spiritual burden with limited formal support [56].

### 5.3. Advancing Research Through Systems-Level, Context-Sensitive and Inclusive Approaches

Future research should move beyond predominantly descriptive accounts toward systems-level inquiry that examines how spiritual care is embedded—or marginalised—within healthcare organisations, care pathways and policy frameworks. Greater methodological precision is needed, particularly regarding disease stage (e.g., advanced versus end-of-life dementia), to better understand how spiritual needs evolve over time. In addition, addressing gaps in the literature requires the inclusion of under-represented perspectives, including healthcare professionals, patients where possible, spiritual care providers and culturally minoritised communities. Such approaches would support more equitable, context-sensitive models of spiritual care and reduce the risk of reproducing culturally narrow understandings of spirituality in neurodegenerative caregiving [6,26,49].

While the findings of this review highlight the need for more structured and system-level approaches to spiritual care for caregivers, the implementation of such responses is not without complexity. Addressing caregivers’ needs within healthcare systems raises important organisational and ethical considerations, including how caregivers are recognised within care pathways, how support is delivered without over-medicalising their role, and how services can be integrated without creating additional burden or fragmentation. These challenges underline the importance of developing context-sensitive and flexible models of care and suggest that future research should explore not only the effectiveness of interventions, but also their feasibility, acceptability, and ethical implications in real-world settings.

In addition, the findings suggest that the needs of different caregiving actors may require distinct approaches. While this review adopts a unifying perspective by considering individuals involved in caregiving roles, in practice the needs of family caregivers and healthcare professionals are likely to be addressed through different organisational pathways. Family caregivers often require informal, relational, and psychosocial support, whereas healthcare professionals may benefit from structured training, supervision, and organisational support systems. Recognising these differences is essential for the development of appropriate and effective models of care [63,64].

These considerations also raise questions regarding the formalisation of caregivers’ involvement in care processes. In particular, if caregivers are to be supported through structured interventions, issues such as voluntary participation, informed engagement, and the boundaries of care provision may need to be carefully considered. While caregivers are not patients in the traditional sense, their increasing visibility within care pathways may require new frameworks to ensure that support is offered in an appropriate, ethical, and non-intrusive manner.

Furthermore, the potential documentation of caregivers’ needs and experiences within healthcare systems raises additional considerations related to confidentiality, data protection, and the appropriate management of sensitive information. As caregivers are not formally recognised as patients, the extent to which their experiences should be recorded, and how such information should be integrated into existing health records, remains an open question. These issues highlight the need for careful reflection on the boundaries between patient-centred and caregiver-centred information within clinical practice.

Finally, the integration of caregivers’ spiritual needs into care processes also raises the possibility of divergent priorities between patients and caregivers. In some situations, what is perceived as beneficial or meaningful for caregivers may not fully align with the preferences or best interests of the patient. These potential tensions highlight the need for careful, ethically informed approaches that recognise the relational nature of care while maintaining clarity about roles, responsibilities, and decision-making processes. Rather than assuming alignment, future research and policy development should explore how such differences can be navigated transparently and respectfully within clinical practice.

## 6. Review Limitations and Dementia Research Bias

This scoping review has several limitations inherent to the methodology and to the nature of the existing evidence base. Consistent with scoping-review guidance, no formal appraisal of methodological quality was undertaken; findings should therefore be interpreted as descriptive and exploratory rather than evaluative. Another limitation relates to the lack of consistent and clearly defined conceptualisations of spirituality across studies, which reflects broader ambiguity in the field and may affect comparability and interpretation of findings. Further limitations emerged during the screening and eligibility phase, particularly in relation to dementia. Although a large number of studies addressed dementia caregiving and, in some cases, referred to spirituality, many did not clearly specify disease stage or situate participants within advanced or explicitly palliative or end-of-life contexts. As a result, these studies could not be included based on the predefined inclusion criteria. This pattern suggests that while dementia is a broad and extensively researched area, studies specifically examining spiritual needs at the end of life in dementia remain comparatively limited, especially in relation to caregiving. The lack of clarity regarding disease trajectory constrained the inclusion of potentially relevant literature and limited the ability to draw conclusions about spiritual needs unique to the terminal phase. In addition, language restrictions and the predominance of studies from the Global North may have limited the inclusion of culturally diverse perspectives. Another limitation relates to the uneven representation of populations, with a strong predominance of family caregivers and limited evidence on healthcare professionals.

## 7. Research Team Positionality Statement

The team brings diverse personal and professional perspectives to the study of spiritual care. Most members identify with a Catholic cultural background, while the lead author identifies as non-religious. We recognise that both religious and non-religious standpoints may shape how spirituality is understood and interpreted, particularly in a field where spirituality, religiosity and existential meaning are often intertwined. These differences were treated as a resource for reflexive dialogue throughout the review. Acknowledging positionality is essential, as it informs epistemological assumptions and interpretive lenses. To mitigate potential bias, the team engaged in ongoing reflexive discussion during study selection, data charting and synthesis, remaining attentive to multiple conceptualisations of spirituality, including secular, relational and existential perspectives alongside religious understandings.

## 8. Conclusions

This scoping review maps how spirituality is understood, experienced and addressed in caregiving for end-stage neurodegenerative disease, encompassing family members, relatives and healthcare professionals as caregivers. Across the literature, spirituality emerged as a dynamic, relational and context-dependent dimension of caregiving; however, caregivers’ spiritual needs and processes were described far more frequently than they were systematically recognised or supported within healthcare systems. Persistent gaps—including conceptual ambiguity, limited attention to support for family caregivers, and cultural imbalances—highlight structural shortcomings in current approaches to spiritual care. Taken together, these findings indicate the need for integrated, caregiver-inclusive and culturally responsive models of spiritual care, with clear implications for the development of educational interventions for healthcare professionals, palliative care services, and for broadening future research to more fully address this context.

## Figures and Tables

**Table 1:** Summary of the studies’ characteristics.

Author and Year	Country	Study Design	Aim	Instrument	Population Type	Context
Brandstötter et al., (2025) [49]	Germany	Quantitative study	To investigate spiritual needs in people with Amyotrophic Lateral Sclerosis (pALS) and their closest caregivers	Spiritual Needs Questionnaire	52 family caregivers	Specialised home-based care
Hoffstädt et al., (2025) [51]	The Netherlands	Quantitative study	to gain understanding of family caregivers’ experiences during the last week of life of their relative with dementia in a nursing home and the support they received	Toolkit of Instruments to Measure End-of-life Care	165 family caregivers	Psychogeriatric unit
Piamjariyakul et al., (2024) [37]	USA	Quantitative study	To determine the factors impacting the spiritual well-being of caregivers	FACIT-Sp-12 questionnaire.	20 family caregivers	Rural Appalachian home-care setting
Aoun et al., (2024) [54]	Australia	Mixed-methods study	To assess the feasibility and impact of the Carers’ Alert Thermometer (CAT).	Carers’ Alert Thermometer (CAT).	30 family caregivers	Home care or hospital clinical
Maksymowicz-Śliwińska et al., (2023) [50]	Germany and Poland	Quantitative study	to analyze the caregivers’ attitudes towards life-prolonging and life-shortening methods in the advanced stage of ALS.	questionnaire	164 Family caregivers	Specialized neurological centers.
Koljack et al., (2022) [47]	USA and Canada	Quantitative study	to evaluate the association between patient and caregiver characteristics relevant within a palliative care framework and overall caregiver spiritual well-being	FACIT-Sp-12 questionnaire.	183 Family caregivers	academic medical centers
Costa et al., (2021) [57]	Brazil (State of Alagos)	Qualitative study	To understand the existential transformations of the family caregiver of a person living with Amyotrophic Lateral Sclerosis.	Phenomenological interview	12 Family caregivers	Health Department
Durepos et al. (2021) [48]	Canada	Mixed-methods study	To develop a multi-dimensional questionnaire titled ‘Caring Ahead’ to assess feelings of preparedness for end-of-life in family caregivers	Caring Ahead’ questionnaire	16 Family caregivers	Long-term care homes
Baumgardner & Mayo, (2021) [15]	USA	Qualitative study	To explore the lived experiences of spiritual well-being amongst family caregivers to persons with dementia receiving palliative care and living at home.	Phenomenological interview	10 Family caregivers	Palliative home care
McLennon et al., (2021) [38]	USA	Qualitative study	to identify the End-of-Life needs, concerns, and advice of family caregivers	narratives	6 Family caregivers	publicly accessible caregiver blogs
Macchi et al., (2020) [39]	USA	Quantitative study	To assess patient and caregiver characteristics, including spirituality and grief, which affect caregiver burden in a palliative population	FACIT-Sp-12 questionnaire.	175 Family caregivers	community-based, outpatient, and residential.
Jensen et al., (2020) [40]	USA	Qualitative study	To identify characteristics of resilience in bereaved caregivers	survey with open question	19 Family caregivers	home care or hospice
Warrier et al., (2020) [59]	India	Qualitative study	The objective was to explore the lived experience of spouses of persons diagnosed with motoneuron disease (MND)	in-depth interview	2 Family caregivers	Care center for neurological disorders
Hovland & Kramer, (2019) [41]	USA	Qualitative study	Investigate the experiences of family caregivers and explored barriers and facilitators to preparation for the family member’s death	in-depth interview	36 Family caregivers	community-based, outpatient, and residential.
Dekawaty et al., (2019) [58]	Indonesia	Qualitative study	explore caregivers’ experiences in caring for family members with Parkinson’s disease.	in-depth interview	5 Family caregivers	community-based, outpatient, and residential.
Steinhauser et al., (2016) [42]	USA	Mixed-methods study	administer and evaluate a standardized chaplain-led intervention to improve the well-being of caregivers	FACIT–Sp, the 5-item anxiety subscale from the modified	26 Family caregivers	outpatient palliative care and ALS clinics
Ozanne et al., (2015) [56]	Sweden	Qualitative study	to illuminate experiences of finding meaning in life	Interviews semi structured	13 Family caregivers	community-based, outpatient, and residential.
Van der Steen et al., ( 2014) [52]	The Netherlands	Quantitative study	To explore how to support the physician’s role in the spiritual caregiving at the end of life	survey	88 Healthcare professionals	Nursing home facilities
Slape (2014) [55]	Australia	Qualitative study	identify spiritual needs of family members during dementia and palliative care stages	in-depth interview	10 Family caregivers	aged care facility
Gijsberts at al., (2013) [53]	The Netherlands	Qualitative study	Investigate: - how spiritual needs are assessed and spiritual care is provided to Dutch nursing home residents; - how caregivers (physicians, nurses, and other staff) communicate and collaborate in addressing residents’ spiritual needs	on-unit observations, informal dialogues, participation in formal clinical meetings, review of care documentation, reflective field notes, and supplementary formal interviews	Family caregivers	Urban nursing home
Cumming (2012) [43]	USA	Quantitative study	to use a variety of instruments to assess the relationship between traits that the caregiver comes into the caring situation with (resiliency, social support, spirituality, hope) and caregiver burden, within different caregiving populations.	Systems of Belief Inventory (SBI15R); Herth Hope Index (HHI); The 14-Item Resilience Scale (RS-14).	95 Family caregivers	different associations in Colorado
DeMond (2009) [44]	USA	Mixed-methods study	The development and implementation of a spiritual-care support group model for family caregivers	24-item Caregiver’s Pastoral Care Assessment questionnaire, interview and Systematic clinical observations	24 Family caregivers	long-term care (LTC) facilities
Sanders et al., (2008) [45]	USA	Mixed-methods study	Describe the lived experience of 44 spouses and adult children who are caregivers	MM-CGI-SF, Brief COPE, Hogan Checklist, FAST	44 Family caregivers	community-based, outpatient, and residential.
Hebert et al., (2007) [46]	USA	Quantitative study	To assess the relationship between several dimensions of religion and mental health in a sample of caregivers	Center for Epidemiologic Studies Depression Scale (CES-)	1229 Family caregivers	community-based, outpatient, and residential.

**Table 2:** Analytical domains, themes and interconnections.

Analytical Domain	Thematic Areas	Analytic Focus	Interconnections
Spiritual needs	1. Nature and dimensions of spiritual needs	Existential, relational and, where relevant, religious needs, including meaning, hope, peace, identity, connection, rituals and transcendence across the caregiving trajectory	Closely linked to spiritual processes through distress, coping and meaning-making
Spiritual processes	2. Spiritual challenges and sources of distress 3. Spirituality as a coping and transformative resource 4. Factors associated with spiritual well-being	Spirituality as a dynamic process shaped by vulnerability, adaptation and transformation, and by the interaction of distress, coping, resilience, relational factors and clinical context	Mediates between needs and care; shaped by support or lack thereof
Spiritual care	5. Spiritual care interventions and support strategies 6. Barriers and gaps in spiritual care provision 7. Contextual influences on spiritual needs and care	Organisational, professional and contextual responses to spiritual needs, including interventions, structural barriers, and the influence of cultural, ethical and service-level factors	Influences both needs and processes through access, recognition and support

## Data Availability

No new data were created or analyzed in this study.

## Supplementary Materials

The following supporting information can be downloaded at: https://www.mdpi.com/article/10.3390/brainsci16060611/s1, Supplementary Material S1: Full Search Strategies for All Databases.
