# Supplementary material for: Spiritual Care Needs and Challenges Among Caregivers and Families of People with Neurodegenerative Diseases in Palliative and End-of-Life Care: A Scoping Review
Source: Brain Sci. 2026 Jun 4;16(6):611. doi: 10.3390/brainsci16060611 (PMC13296635; doi:10.3390/brainsci16060611)
Supplement: Supplementary file 1 [file brainsci-16-00611-s001.zip › brainsci-4270486-supplementary.pdf]

Supplementary Material S1. Full Search Strategies for All Databases

DATA BASE	**PUBMED**	
	**POPULATION**	**RESULT**
#1	("Health Personnel"[Mesh]) OR ("Healthcare workers"[Title/Abstract] OR "Healthcare staff"[Title/Abstract] OR Nurse*[Title/Abstract] OR Physician*[Title/Abstract]) OR ("Family"[Mesh]) OR ("Caregivers"[Mesh]) OR (Relative*OR Caregiver*OR "family caregivers"[Title/Abstract])	
	**CONCEPT**	
#2	("Spirituality"[Mesh]) OR (spirituality [Title/Abstract] OR "spiritual sensitivity"[Title/Abstract] OR spiritual [Title/Abstract] OR “Spiritual Care” [Title/Abstract] OR “Spiritual Distress” [Title/Abstract] OR “Spiritual Need” [Title/Abstract])	
	**CONTEXT**	
#3	("Neurodegenerative Diseases"[Mesh]) OR ("Motor Neuron Disease"[Mesh]) OR ("Neuromuscular Diseases"[Mesh]) OR ("Parkinson Disease"[Mesh]) OR ("Dementia"[Mesh]) OR (neurodegenerative[Title/Abstract] OR amyotrophic[Title/Abstract] OR Parkinson[Title/Abstract] OR dementia[Title/Abstract] OR "neurologic disorder"[Title/Abstract])	
	#1 AND #2 AND #3	277

DATA BASE	**EBSCOhost-CINAHL Plus with Full Text**	
	**POPULATION**	**RESULT**
#1	XB MM "Health Personnel+" OR XB ("healthcare workers" OR "healthcare staff" OR "healthcare professionals" OR nurse* OR Physician*) OR XB MM "Family+" OR XB MM "Caregivers" OR XB (caregivers or family members or relatives or informal caregivers)	
	**CONCEPT**	
#2	XB MM "Spirituality" OR XB (spirituality OR "spiritual sensitivity" OR spiritual OR “Spiritual Care” OR “Spiritual Distress” OR “Spiritual Need")	
	**CONTEXT**	
#3	MM "Motor Neuron Diseases+" OR XB MM "Neurodegenerative Diseases+" OR XB MM "Parkinson Disease" OR XB MM "Dementia+" OR XB (neurodegenerative OR amyotrophic OR Parkinson OR dementia OR "neurologic disorder")	
	#1 AND #2 AND #3	211

DATABASE	**EBSCOhost- APA PsycInfo**	
	**POPULATION**	**RESULT**
#1	XB (DE "Health Personnel" OR DE "Allied Health Personnel" OR DE "Clinicians" OR DE "Direct Care Workers" OR DE "Medical Personnel" OR "healthcare workers" OR "healthcare staff" OR "healthcare professionals" OR nurse* OR Physician* OR DE "Family" OR DE "Caregivers" OR caregivers OR family members OR relatives OR "informal caregivers")	
	**CONCEPT**	
#2	XB (DE "Spirituality" OR DE "Spiritual Well Being" OR spirituality OR "spiritual sensitivity" OR spiritual OR “Spiritual Care” OR “Spiritual Distress” OR “Spiritual Need")	
	**CONTEXT**	
#3	XB (DE "Nervous System Disorders" OR DE "Neurodegenerative Diseases" OR DE "Neurofibromatosis" OR DE "Neuroinflammation" OR DE "Neuromuscular Disorders" OR DE "Sclerosis (Nervous System)" OR DE "Dementia" OR neurodegenerative OR amyotrophic OR Parkinson OR dementia OR "neurologic disorder")	
	#1 AND #2 AND #3	271

DATABASE	**SCOPUS**	
	**POPULATION**	**RESULT**
#1	TITLE-ABS-KEY ("healthcare workers" OR "healthcare staff" OR "healthcare professionals" OR nurse* OR physician* OR caregivers OR family AND members OR relatives OR "informal caregivers")	
	**CONCEPT**	
#2	TITLE-ABS-KEY (spirituality OR "spiritual sensitivity" OR spiritual OR "Spiritual Care" OR "Spiritual Distress" OR "Spiritual Need")	
	**CONTEXT**	
#3	TITLE-ABS-KEY (neurodegenerative OR amyotrophic OR parkinson OR dementia OR "neurologic disorder")	
	#1 AND #2 AND #3	133
